# Functional connectivity in default mode network correlates with severity of hypoxemia in obstructive sleep apnea

**DOI:** 10.1002/brb3.1889

**Published:** 2020-11-01

**Authors:** Ya‐Ting Chang, Yung‐Che Chen, Yung‐Lung Chen, Shih‐Wei Hsu, Feng‐Yueh Yang, Chen‐Chang Lee, Po‐Yuan Hsu, Meng‐Chih Lin

**Affiliations:** ^1^ Department of Neurology Kaohsiung Chang Gung Memorial Hospital Chang Gung University College of Medicine Kaohsiung Taiwan; ^2^ Division of Pulmonary & Critical Care Medicine Department of Internal Medicine Kaohsiung Chang Gung Memorial Hospital Chang Gung University College of Medicine Kaohsiung Taiwan; ^3^ Division of Cardiology Department of Internal Medicine Kaohsiung Chang Gung Memorial Hospital Chang Gung University College of Medicine Kaohsiung Taiwan; ^4^ Department of Radiology Kaohsiung Chang Gung Memorial Hospital Chang Gung University College of Medicine Kaohsiung Taiwan

**Keywords:** cognition, neuroimaging

## Abstract

**Introduction:**

Obstructive sleep apnea (OSA)‐associated hypoxemia, sleep fragmentation, and cerebral vascular dysfunction are implicated in cognitive dysfunction. Functional connectivity within default mode network (DMN) is a possible mechanism underlying the cognitive impairment. The aim of this study was to investigate the impact of hypoxemia and sleep fragmentation on functional connectivity and on cognitive performance in patients with OSA.

**Methods:**

Twenty‐eight patients with OSA were included (mean age = 58.0 ± 8.5 years). We correlated the functional connectivity in DMN with cognitive performances and further analyzed the relationship of functional connectivity in DMN with hypoxemia severity, as revealed by apnea‐hypopnea index (AHI), oxygen desaturation index (ODI), and nadir SaO_2_ (%), and with degree of sleep fragmentation, as shown by sleep efficiency and wake after sleep onset.

**Results:**

Functional connectivity in DMN was associated with AHI, ODI, and nadir SaO_2_ (%) (*p* < .05) and was not associated with sleep fragmentation measures (*p* > .05). Functional connectivity that was associated with AHI, ODI, and nadir SaO_2_ (%) was in the areas of bilateral middle temporal gyri, bilateral frontal pole, and bilateral hippocampus and was positively correlated with Cognitive Abilities Screening Instrument (CASI) total score (*ρ* = 0.484; *p* = .012), CASI‐List‐generating, CASI‐Attention, and composite score of CASI‐List‐generating plus CASI‐Attention (*p* < .05).

**Conclusion:**

Functional connectivity in DMN is implicated in impairment of global cognitive function and of attention in OSA patients. The functional connectivity in the DMN is associated with hypoxemia rather than with sleep fragmentation.

## Graphical abstract

The functional connectivity within the DMN was associated with hypoxemia severity, as revealed by apnea‐hypopnea index, oxygen desaturation index, and nadir SaO_2_ (%), rather than with degree of sleep disruption, as shown by sleep efficiency and wake after sleep onset.

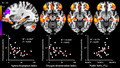

## 1 INTRODUCTION

Obstructive sleep apnea (OSA) is a sleep‐disordered breathing characterized by repeated pauses (apneas) or decreases (hypopneas) in breathing during sleep. OSA‐associated hypoxemia, sleep fragmentation and low sleep efficiency, and cerebral vascular dysfunction (Engleman et al., 2000) potentially affect impairment in global cognition and deficits in memory, attention, and executive performances (Dzierzewski et al., 2018; Olaithe et al., 2018).

Longer total sleep time (TST) (min) and greater wake after sleep onset (WASO) (min) are linked to poorer global cognitive function, worse verbal fluency, and poorer memory in older women (Spira et al., 2017). However, the relationship between objective sleep measures and cognitive performances in these individuals is driven by patients with dementia (Spira et al., 2017). Another study shows that sleep fragmentation (longer minutes of WASO and lower sleep efficiency) is associated with more global cognitive impairment and with more changes of cognitive decline (McSorley et al., 2019). WASO (min) is further shown to moderate the relationship between amyloid‐β accumulation and memory, which suggests that sleep efficiency plays a possible protective role in preclinical stage of dementia (Wilckens et al., 2018).

The profile of cognitive deficits in patients with OSA has been well demonstrated in the reviews and meta‐analyses (Beebe et al., 2003; Saunamaki & Jehkonen, 2007; Wallace & Bucks, 2013). In addition to identifying specific cognitive impairment, elucidating the neural mechanisms of cognitive dysfunction and their relationship with hypoxemia, sleep fragmentation, and the polysomnography (PSG)‐derived objective sleep measures will merit investigation of therapy that may improve cognitive prognosis and ameliorate neurobehavioral impairment in OSA.

Resting state functional magnetic resonance imaging (rs‐fMRI) is a potential biomarkers of relationship between cognitive impairment and OSA, because decreased functional connectivity in the default mode network (DMN) has been shown in patients with OSA (Chen et al., 2018; Li et al., 2016; Yu et al., 2019). In these individuals, connectivity of posterior cingulate cortex (PCC)/precuneus with hippocampus is negatively correlated with memory (Li et al., 2016) and reduced connectivity of prefrontal cortex with amygdala is the possible mechanism underlying affective deficits in men with severe OSA (Yu et al., 2019).

Independent component analysis (ICA) is a data‐driven technique that separates mixed signals to construct distinct component and divides the image data into spatially independent activation maps without any presumed seed regions or volumes of interest. The DMN identified by ICA includes several key hubs including hippocampus, PCC/precuneus, lateral temporal cortex, and prefrontal cortex (Andrews‐Hanna et al., 2010). Here, we first characterized functional connectivity and cognitive performances in patients with OSA. We aimed to evaluate the relationship of functional connectivity with the severity of OSA by correlating the functional connectivity in the DMN and PSG‐derived sleep measures, such as apnea‐hypopnea index (AHI), oxygen desaturation index (ODI), nadir SaO_2_ (%), sleep efficiency, WASO counts, WASO time (min), and TST (min). We hypothesized that the hypoxemia‐related measures (AHI, ODI, and nadir SaO_2_) rather than the sleep fragmentation‐related measures (sleep efficiency and WASO) would influence the functional connectivity, which was associated with cognitive performances.

## 2 MATERIALS AND METHODS

### 2.1 Inclusion and exclusion criteria

Twenty‐eight patients with OSA from outpatient department were enrolled in this study. Participants who met criteria for OSA according to international classification of sleep disorders—third edition (Sateia, 2014)—and had an AHI ≥ 5 were included. Furthermore, OSA patients, with age between 40 and 70 years, had to be treatment free. The exclusion criteria for the study were as follows: (a) education level less than 6 years; (b) congestive heart failure (Inamdar & Inamdar, 2016); (c) uncontrolled diabetes mellitus and hypertension (Petersmann et al., 2018); (d) chronic kidney disease (Snyder & Pendergraph, 2005); (e) chronic obstructive pulmonary disease; (f) BMI more than 35; (g) any abnormalities in thyroid function, vitamin B12 level, and folic acid level; (h) clinical dementia rating (CDR) ≥ 1 or the presence of mild cognitive impairment/dementia syndrome according to the diagnostic and statistical manual of mental disorders, fourth edition (American Psychiatric Association, 1994), and the diagnosis was on the basis of consensus from a panel composed of neurologists, neuropsychologists, and neuroradiologists (Chang, Hsu, et al., 2019); (i) coexisting neurodegenerative disease; (j) alcoholism or substance abuse; and (k) use of respiratory stimulants or depressants.

### 2.2 PSG

At Sleep Center of Kaohsiung Chang Gung Memorial Hospital, all‐night attended comprehensive diagnostic sleep studies were performed. As our previous study (Chang et al., 2017), standardized PSG measured electroencephalography, electrocardiogram, submental electromyography, electrooculography, nasal and oral airflow, oxygen saturation, sleep position, and so on. Sleep stage classification, AHI, ODI, nadir SaO_2_ (%), sleep efficiency, WASO counts, WASO time (min), and TST (min) were all obtained and further analyzed. Daytime sleepiness was assessed using the Epworth Sleepiness Scale (ESS) (Johns, 1991). Sleep fragmentation‐related measures included sleep efficiency, WASO counts, and WASO time (min) (Shrivastava et al., 2014). Lower sleep efficiency, higher WASO counts, and higher WASO time (min) indicated greater sleep fragmentation.

### 2.3 Study design

Cognitive testing and magnetic resonance imaging (MRI) were all performed within duration of 4 weeks. All patients were provided full informed written consents. This study was approved by Chang Gung Memorial Hospital's Institutional Review Committee on Human Research and was in accordance with the 1964 Helsinki Declaration and its later amendments or comparable ethical standards. To find the right amount of air pressure for individual subjects, CPAP titration was scheduled after a physician carefully reviewed the results of the in‐lab sleep study. During the processes, all of the examination would be completed as soon as possible. CPAP treatment was provided as scheduled processes.

### 2.4 MRI acquisition

MRI was acquired on a Siemens 3T scanner. All participants underwent an MRI session for about 40 min. The scanning protocol of T1‐weighted imaging included inversion‐recovery‐prepared, 3‐dimensional, spoiled, gradient‐recalled acquisition in a steady‐state sequence with a repetition time/inversion time of 8,600 ms/450 ms, 240 × 240 mm field of view, and 1‐mm slice thickness. For the rs‐fMRI scan, the participants were requested to avoid sleep deprivation, to avoid caffeine or sedatives 8 hr before the examination, to lie still with closed eyes, not to fall asleep, and not to think about any one thing during the examination. Every participant was asked whether they falling asleep, anxiety, or agitation during the examination, and none of the participants reported they did. None of our patients were under treatment with acetylcholine esterase inhibitors or any other treatment that possibly influenced functional connectivity.

The acquisition and analysis of rs‐fMRI used the same protocol as our previous study (Chang, Hsu, et al., 2019; Chang, Mori, et al., 2019), by using CONN toolbox (http://www.nitrc.org/projects/conn) (Whitfield‐Gabrieli & Nieto‐Castanon, 2012). To remove motion artifacts in rs‐fMRI preprocessing, variations in the average blood oxygen‐level‐dependent signal from scan to scan should be less than 1%. In addition, framewise displacement should be less than 0.25 mm/TR. Simultaneously, images were detrended and filtered to a frequency between 0.008 and 0.09 Hz. Anatomical component‐based noise correction methods, as implemented in the CONN toolbox (http://www.nitrc.org/projects/conn) (Whitfield‐Gabrieli & Nieto‐Castanon, 2012), was used to regress out the head movement time series, white matter, and cerebrospinal fluid signals from each voxel.

### 2.5 The rs‐fMRI ICA

**FIGURE 1:**
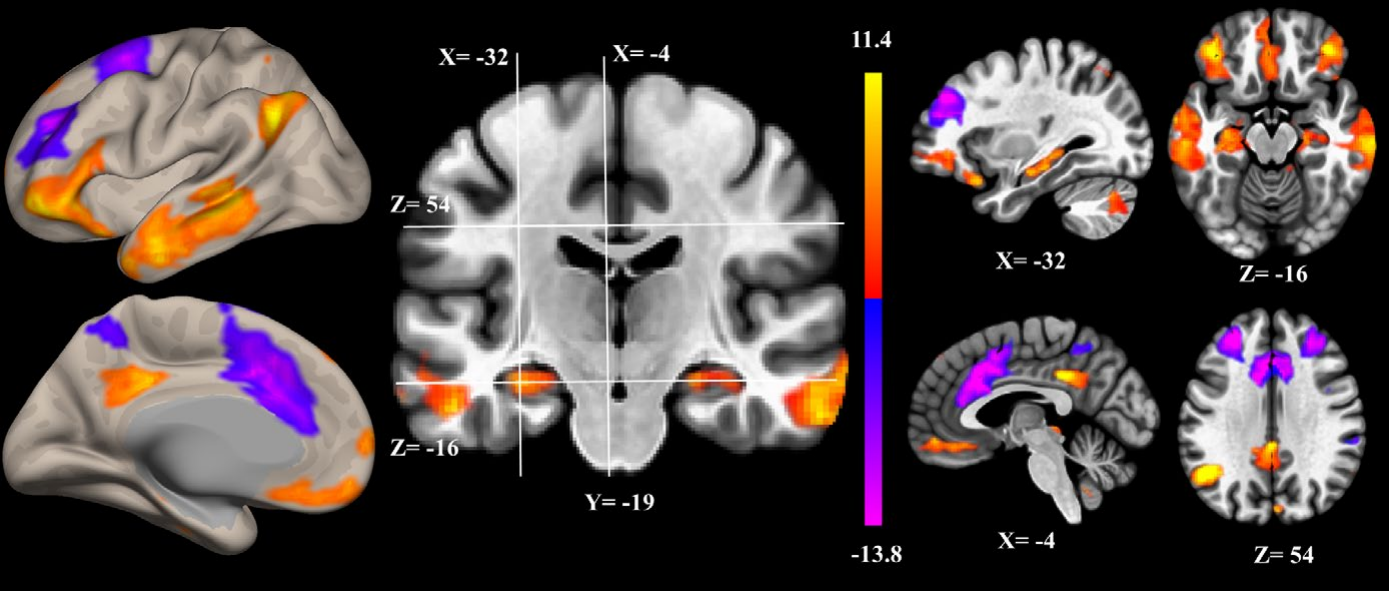
Z‐stat maps of independent component analysis of resting stage‐functional magnetic resonance imaging‐based covariant spatial patterns for default mode network

ICA assumes that the observed variables are a linear combination of a set of statistically independent components (ICs) having non‐Gaussian distribution (Brown et al., 2001). De‐convolution of the observed signal revealed the hidden structure. Spatial ICA‐extracted ICs represented functional networks vary coherently across subjects. The distribution of voxels in each component had been shown to be statistically independent of the distribution in other components (McKeown et al., 1998), delineating the borders between possibly overlying networks (Kalcher et al., 2012). Spatial ICA of the preprocessed fMRI was performed using the CONN toolbox (http://www.nitrc.org/projects/conn) (Whitfield‐Gabrieli & Nieto‐Castanon, 2012). Principal component analysis was used for whitening, reducing the data of each image, and estimating the ICs. The optimal number of components was 20, which was determined from the data using the CONN dimensionality estimation tool, representing a good trade‐off between preservation of the information intrinsically contained in the data and reduction of its size. Spatial ICs with pathophysiological or functional‐anatomic meaning of DMN components are demonstrated in Figure 1 (Andrews‐Hanna et al., 2010). After scaling the parameter estimates of functional connectivity strength to *z*‐values, the resulting spatial maps containing voxel‐wise information about the magnitude of strength of correlation among the nodes were used to extract functional connectivity at the individual subject level (Allen et al., 2012). All analysis used the *z*‐values of functional connectivity strength.

### 2.6 Neuropsychological assessments

Participants were evaluated of general cognitive performance using CDR (Morris, 1993) and Cognitive Abilities Screening Instrument (CASI) (Liu et al., 1994). CDR was used to rate performances in six domains, including memory, judgment and problem solving, community affairs, home and hobbies, orientation, and personal care. In addition to general cognitive performance, CASI was used to evaluate specific domains, including short‐term memory (score range from 0 to 12), long‐term memory (score range from 0 to 10), orientation (score range from 0 to 18), attention and concentration (score range from 0 to 8), abstraction (score range from 0 to 12), visual construction (score range from 0 to 10), language (score range from 0 to 10), and list‐generating fluency (score range from 0 to 10) (Meguro et al., 2001). CASI was an objective test, and it could be administered in 15–20 min. The nine domain scores added up to a total score, ranging from 0 to 100. Attention and concentration domain consisted of the items of repeating three words and repeating digits backward. List‐generating fluency consisted of the items of generating names of four‐legged animals in 30 s. The two domain scores added up to a composite score of CASI‐List‐generating plus CASI‐Attention.

### 2.7 Statistical analysis

Clinical data were expressed as mean ± standard deviation. Independent *t*‐tests were used to compare continuous variables between patients with mild to moderate OSA (AHI < 30) and those with severe OSA (AHI ≥ 30) (Kushida et al., 2001). We used Spearman's rank correlation to measure the relationship between the neuroimaging data and evaluated the effect from AHI, ODI, and nadir SaO_2_ (%). After controlling for age and educational level, partial correlation was used to analyze the relationship between the cognitive scores and neuroimaging data. All statistical analyses were conducted using SPSS software (SPSS version 22 for Windows®, SPSS Inc., Chicago, IL). Statistical significance was set at *p* < .05.

## 3 RESULTS

### 3.1 Demographic data

Twenty‐eight patients with OSA completed the study (Table 1). Fourteen patients had mild to moderate OSA, and the other fourteen patients had severe OSA. The age (years), education level (years), ESS, sleep efficiency, WASO counts, WASO time (min), TST (min), CDR, rapid eye movement (REM) (%), all subscores of CASI, and CASI total score were not significantly different between the two groups (*p* > .05). The severe OSA group had significant higher ODI (*t*(27) = 4.420; *p* < .001) and lower nadir SaO_2_ (*t*(27) = −2.449; *p* = .021) than mild to moderate OSA group did (Table S1).

**TABLE 1:** General characteristics of the patients with obstructive sleep apnea

Characteristic	Mean ± standard deviation
Sample size (*n*)	28
Age (years)/education (years)	58.0 ± 8.5/13.1 ± 3.1
Sex (female/male)	12/16
Epworth Sleepiness Scale	10.5 ± 6.1
Sleep efficiency/TST (min)	0.76 ± 0.19/277.6 ± 51.0
WASO counts/WASO time (min)	21.3 ± 14.3/48.8 ± 39.1
Rapid eye movement (%)	14.4% ± 8%
Apnea‐hypopnea index	39.2 ± 25.5
Oxygen desaturation index	28.4 ± 21.9
Nadir SaO2 (%)	80% ± 8%
CASI total score	92.6 ± 5.9
CASI‐Abstraction	10.1 ± 1.7
CASI‐Attention	7.1 ± 1.0
CASI‐Concentration	9.3 ± 1.2
CASI‐Language ability	9.7 ± 0.5
CASI‐List‐generating	8.5 ± 2.0
CASI‐Long‐term memory	9.9 ± 0.4
CASI‐Orientation	17.7 ± 1.0
CASI‐Short‐term memory	10.4 ± 1.6
CASI‐Visual construction	9.9 ± 0.4

Note

Parametric continuous variables presented as mean ± standard deviation.

Abbreviations: CASI, Cognitive Abilities Screening Instrument; TST, total sleep time; WASO, wake after sleep onset.

### 3.2 The influence of severity of OSA on functional connectivity

**FIGURE 2:**
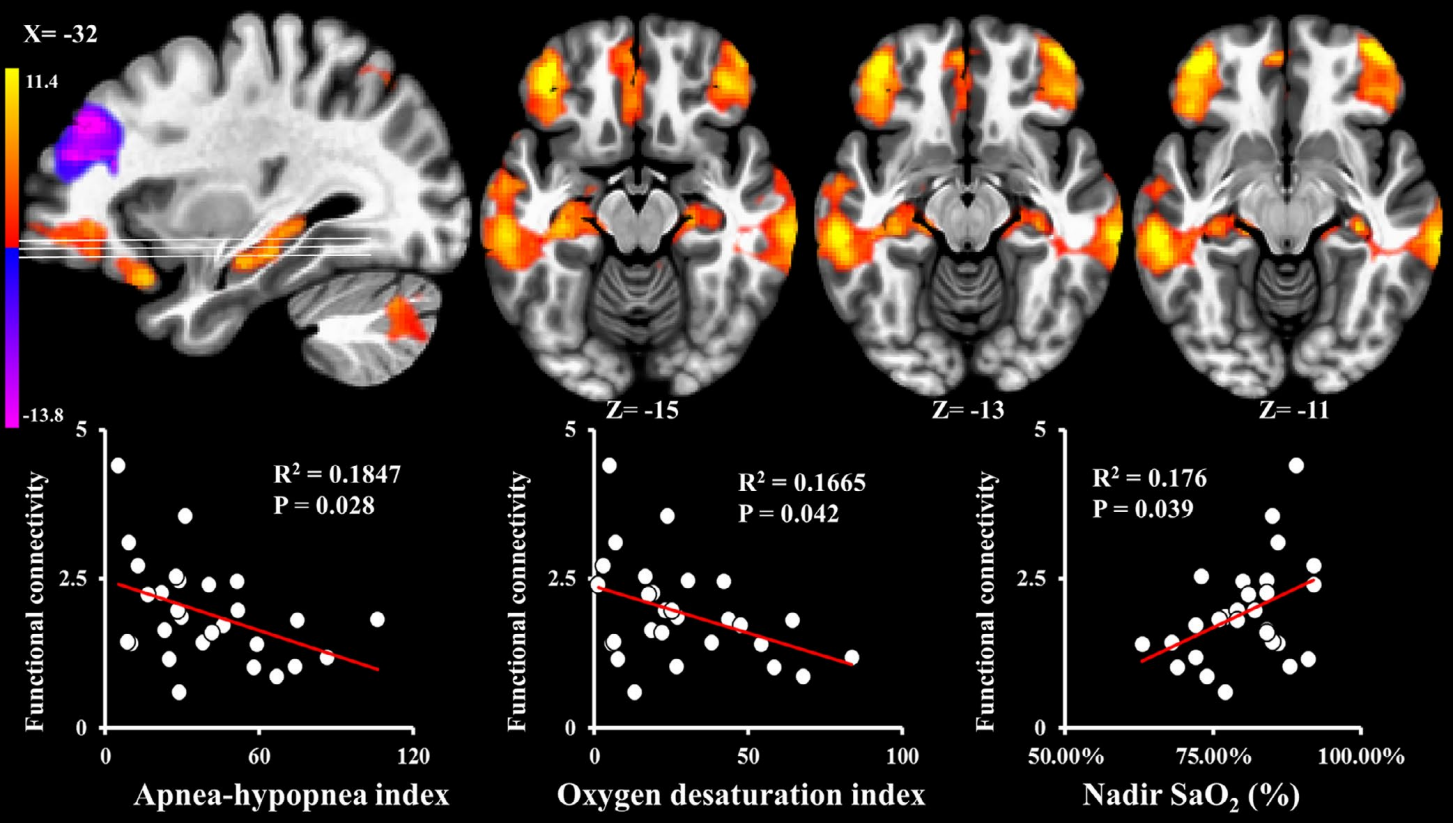
Z‐stat maps of independent component analysis of resting stage‐functional magnetic resonance imaging‐based covariant spatial patterns for bilateral middle temporal gyri, bilateral frontal pole, and bilateral hippocampus (upper row) within default mode network and the correlation of functional connectivity strength (*z*‐values) in the regions shown in the upper row with polysomnography measures (lower row)

Functional connectivity within the DMN (Figure 1) was negatively correlated with AHI (*ρ* = −0.414; *p* = .028; Figure 2) and ODI (*ρ* = −0.388; *p* = .042; Figure 2) and positively correlated with nadir SaO_2_ (*ρ* = 0.393; *p* = .039; Figure 2), and was not associated with sleep efficiency, WASO counts, WASO time (min), TST (min), REM (%), and ESS (*p* > .05). After adjusting for multiple comparisons, only AHI was significantly associated with functional connectivity within the DMN. Neural activity that was associated with AHI, ODI, and nadir SaO_2_ was in the areas of bilateral middle temporal gyri, bilateral frontal pole, and bilateral hippocampus (upper row of Figure 2). PCC/precuneus neural activity within DMN was not associated with AHI, ODI, nadir SaO_2_, sleep efficiency, WASO counts, WASO time (min), TST (min), REM (%), and ESS (*p* > .05). CASI total score was not associated with any hypoxemia‐related or sleep fragmentation‐related measures (*p* > .05).

### 3.3 Spatial ICA map and its clinical significance

**FIGURE 3:**
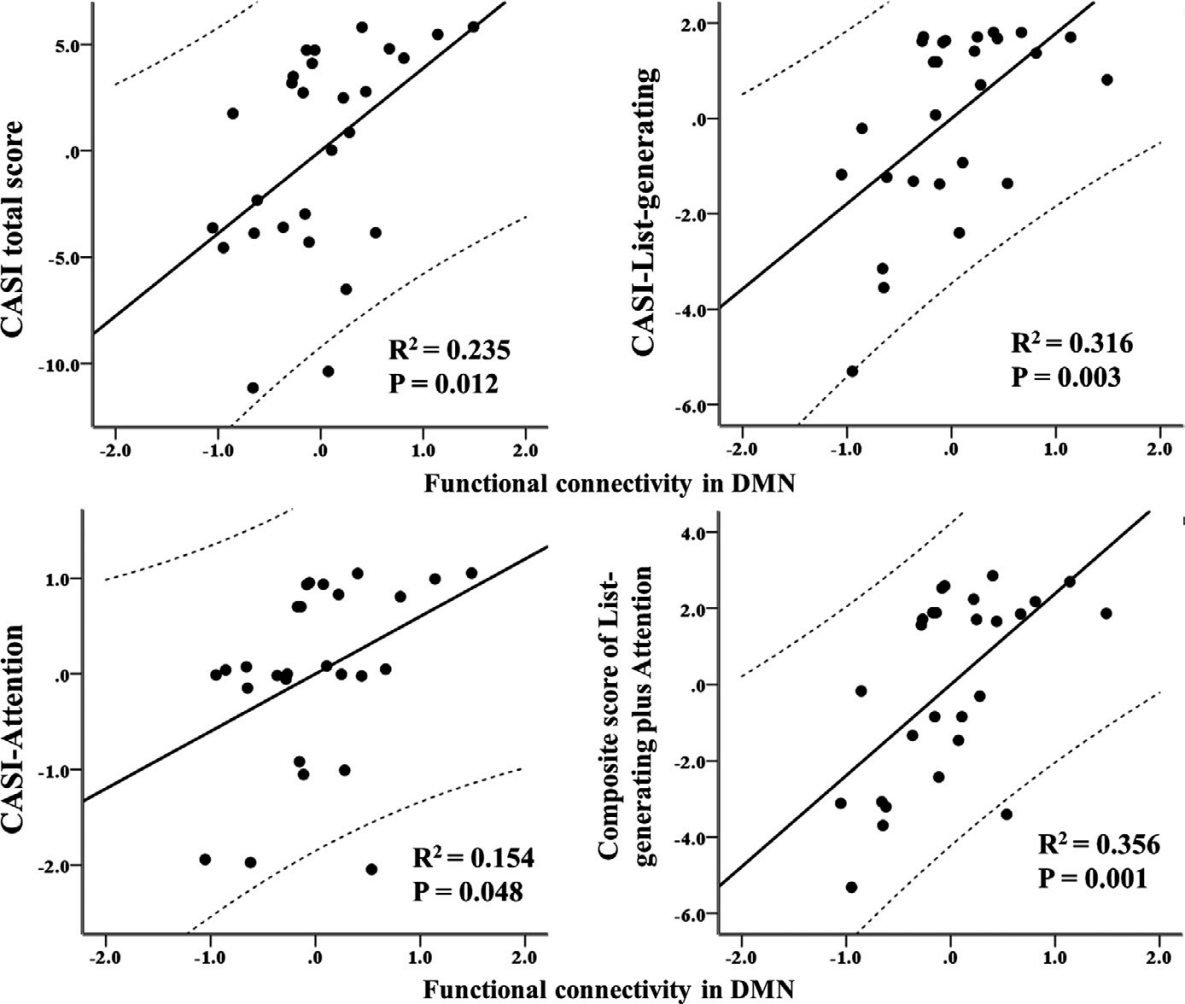
The correlation between functional connectivity in default mode network (DMN) that is associated with each cognitive score. Residuals are plotted to determine the correlation between each variable. The 95% confidence interval is the area enclosed by the dashed curves. CASI, Cognitive Abilities Screening Instrument

The functional connectivity within the DMN that was associated with AHI, ODI, and nadir SaO_2_ was positively correlated with CASI total score (*ρ* = 0.484; *p* = .012; Figure 3), CASI‐List‐generating (*ρ* = 0.562; *p* = .003; Figure 3), CASI‐Attention (*ρ* = 0.392; *p* = .048; Figure 3), and composite score of CASI‐List‐generating plus CASI‐Attention (*ρ* = 0.596; *p* = .001; Figure 3). After adjusting for multiple comparisons, composite score of CASI‐List‐generating plus CASI‐Attention (*p* = .001), CASI‐List‐generating score (*p* = .003), and CASI total score (*p* = .012) were still significantly positively correlated with functional connectivity within the DMN. Other subscores did not associate with any functional connectivity strength of DMN (*p* > .05). Other regional neural activity within DMN did not associate with any cognitive scores (*p* > .05).

In patients with severe OSA, the functional connectivity within the DMN that was associated with AHI, ODI, and nadir SaO_2_ was still positively correlated with CASI total score (*ρ* = 0.653; *p* = .016), CASI‐List‐generating (*ρ* = 0.614; *p* = .026), CASI‐Attention (*ρ* = 0.599; *p* = .030), and composite score of CASI‐List‐generating plus CASI‐Attention (*ρ* = 0.706; *p* = .007). After adjusting for multiple comparisons, composite score of CASI‐List‐generating plus CASI‐Attention (*p* = .007) and CASI total score (*p* = .016) were still significantly positively correlated with functional connectivity within the DMN. However, in patients with mild to moderate OSA, the functional connectivity within the DMN did not associate with any cognitive scores as described above (*p* > .05).

## 4 DISCUSSION

### 4.1 Main findings

In the present study, we implemented ICA with rs‐fMRI to investigate the effect of the OSA severity on functional connectivity in DMN. There were two main findings. First, hypoxemia‐related measures rather than sleep fragmentation‐related measures were linked with functional connectivity in DMN and with neural activity in areas of bilateral middle temporal gyri, bilateral frontal pole, and bilateral hippocampus, whereas neural activity in PCC/precuneus was not affected by any objective sleep measures. The functional connectivity that was associated with hypoxemia‐related measures was associated with CASI total score and subscores in list‐generating, attention, and composite score of list‐generating plus attention.

### 4.2 Detrimental effect of hypoxemia on the functional connectivity in the DMN

Our OSA study demonstrated a negative correlation of functional connectivity within DMN with AHI and ODI. A previous study has demonstrated that patients with OSA have decreased functional connectivity between hippocampus and PCC/precuneus than good sleepers do (Li et al., 2016). In agreement with the study, we further showed that higher AHI, higher ODI, and lower nadir SaO_2_ were associated with lower neural activity in bilateral lateral temporal and hippocampus regions typically involved with DMN. The pathological mechanism underlying the decreased neural activity of hippocampus is associated with a reduced cerebrovascular reactivity of the hippocampus, which is induced by prolonged nocturnal hypoxemia (Prilipko et al., 2014). Another possible mechanism underlying reduced functional connectivity is that higher AHI has been shown to be associated with increased white matter hyperintensities (Del Brutto et al., 2017), and the increased white matter damage has been demonstrated to affect functional connectivity (Sharp et al., 2011). In addition to changes in functional connectivity, structural abnormality of hippocampus and bilateral temporal lobes have been demonstrated in OSA patients, who are at risk for dementia (Cross et al., 2018). Taken together, functional and structural changes in hippocampus and temporal lobes are associated with hypoxemia in OSA.

Using seed‐based functional connectivity analysis to correlate hypoxemia‐related measures with neural activity in DMN, previous studies have not demonstrated any direct relationship between functional connectivity and hypoxemic severity in OSA patients (Li et al., 2016; Yu et al., 2019). Nonetheless, we demonstrated the correlation of functional connectivity in DMN with AHI, ODI, and nadir SaO_2_. It is possibly because of the differences in analysis methods of functional connectivity. This present study used a data‐driven approach of ICA rather than used a hypothesis‐driven approach of seed‐based correlation, and we showed the adverse impact of hypoxemic severity on functional connectivity in DMN. Moreover, our results suggested that both the duration of hypoxemia (ODI and AHI) and the severity of intermittent hypoxemia (nadir SaO_2_) were important factors underlying DMN dysfunction in OSA.

### 4.3 Cognitive correlation of functional connectivity in the DMN

ICA has been used to extract covariance of neuronal activity of interconnected regions involved in the DMN. ICA of rs‐fMRI has been functionally constructed into independent covariance patterns (Damoiseaux et al., 2012). Reduced functional connectivity in the DMN has been associated with memory dysfunction in patients with OSA (Li et al., 2016). The neural activity not only is involved with memory but is also implicated in other cognitive functions (Buckner et al., 2008). The DMN plays a role in supporting a broad low‐level focus of attention to monitor the external environment (Gilbert et al., 2007; Mason et al., 2007). The neural activity of DMN is also negatively correlated with speed of response (Sharp et al., 2011). In the present study of OSA, we showed that functional connectivity in the DMN is associated with out‐of‐scanner cognitive performances, including global cognition, list‐generating, attention, and composite score of list‐generating plus attention, which emphasize the influence of the DMN on impairment of multiple cognitive domains in OSA.

### 4.4 Limitations

There are several limitations to this study. First, it is a cross‐sectional study. Further longitudinal studies are needed to elucidate the impact of hypoxemia on decreased functional connectivity in DMN. Therapy for improvement of DMN functional connectivity may facilitate the recovery of cognitive function after adequate treatment for OSA. Second, the fact that our subjects' CDR was <1 restricted the generalization of our results to individuals with OSA and moderate to severe cognitive impairment. Third, cognitive performance was not conducted with more scientific rigor instruments, such as episodic memory with SRT or AVLT, and Trails A and B. Fourth, the findings of functional connectivity were non‐task‐based fMRI. Fifth, there was no control group for this study. Therefore, this study's conclusions might and might not reflect on the pathological level of OSA on brain function. Including non‐OSA controls will be needed in the future to further investigate the pathological changes of brain function in OSA.

## 5 CONCLUSION

First, our results provided evidence to prove the relationship between functional connectivity in the DMN and hypoxemic severity in OSA. We showed that functional connectivity within the DMN is associated with hypoxemia rather than with sleep fragmentation. Second, the functional connectivity in DMN was implicated in global cognition and several cognitive subdomains in OSA patients. Our results emphasized the importance of studying the influence of hypoxemia on changes in the DMN and suggested that the treatment for improvement of hypoxemia in patients with OSA should be further studied in the context of cognitive function improvement.

## CONFLICT OF INTEREST

The authors declare that they have no conflict of interest. All of the authors did not have financial or other interest in the product or distributor of the product. There was no kind of associations, such as consultancies, stock ownership, or other equity interests or patent‐licensing arrangements, between the authors and the manufacturer or distributor of the product.

## AUTHOR CONTRIBUTIONS

Ya‐Ting Chang: (1) Research project: A. Conception, B. Organization, C. Execution; (2) Statistical Analysis: A. Design, B. Execution, C. Review and Critique; (3) Manuscript: A. Writing of the first draft, B. Review and Critique. Yung‐Che Chen: (1) Statistical Analysis: A. Design, B. Execution, C. Review and Critique; (2) Manuscript: A. Review and Critique. Yung‐Lung Chen: (1) Research project: A. Execution. Shih‐Wei Hsu: (1) Research project: A. Execution. Feng‐Yueh Yang: (1) Research project: A. Execution. Chen‐Chang Lee: (1) Research project: A. Execution. Po‐Yuan Hsu: (1) Research project: A. Execution. Meng‐Chih Lin: (1) Research project: A. Conception, B. Organization, C. Execution; (2) Manuscript: A. Review and Critique. All authors read and approved the final manuscript.

## ETHICAL APPROVAL

The study was approved by Chang Gung Memorial Hospital's Institutional Review Committee on Human Research (201801944B0).

## RESEARCH INVOLVING HUMAN PARTICIPANTS

All procedures performed in studies involving human participants were in accordance with the ethical standards of the institutional and/or national research committee and with the 1964 Helsinki Declaration and its later amendments or comparable ethical standards.

## INFORMED CONSENT

Informed consent was obtained from all individual participants included in the study.

### Peer Review

The peer review history for this article is available at https://publons.com/publon/10.1002/brb3.1889.

## Supporting information

Table S1

## Data Availability

The datasets used and/or analyzed during the current study are available from the corresponding author on reasonable request.
