# Supplementary material for: Functional connectivity in default mode network correlates with severity of hypoxemia in obstructive sleep apnea
Source: Brain Behav. 2020 Nov 1;10(12):e01889. doi: 10.1002/brb3.1889 (PMC7749584; doi:10.1002/brb3.1889)
Supplement: Supplementary file 1 — Table S1 [file BRB3-10-e01889-s001.docx]

**Supplementary Table 1. General characteristics of the patients with mild to moderate obstructive sleep apnea (OSA) and those with severe OSA**

|  | Mean ± standard deviation | |  |
| --- | --- | --- | --- |
| Characteristic | Mild to moderate  OSA group | Severe  OSA group | P vale |
| Sample size (n) | 14 | 14 |  |
| Age (years) | 55.9±8.4 | 60.1±8.4 | 0.204 |
| Education (years) | 12.1±3.2 | 14.2±2.7 | 0.067 |
| Sex (Female/Male) |  |  |  |
| Epworth Sleepiness Scale | 10.3±6.4 | 10.7±6.0 | 0.866 |
| Sleep efficiency | 0.7±0.2 | 0.8±0.1 | 0.143 |
| WASO counts | 18.3±13.0 | 24.4±15.4 | 0.270 |
| WASO time (minutes) | 55.4±50.7 | 42.1±22.8 | 0.378 |
| Apnea-Hypopnea Index | 19.6±8.9 | 58.9±20.9 | <0.001 |
| Oxygen Desaturation Index | 14.3±9.0 | 42.5±22.1 | <0.001 |
| Nadir SaO2 (%) | 0.8±0.1 | 0.8±0.1 | 0.021 |
| CASI Total Score | 92.5±6.7 | 92.7±5.2 | 0.950 |

Parametric continuous variables presented as mean ± standard deviation. CASI, Cognitive Abilities Screening Instrument; WASO, wake after sleep onset.
